# An isotype-specific phosphorylation of Hsp90 rewires co-chaperone regulations

**DOI:** 10.1016/j.jbc.2026.113292

**Published:** 2026-06-23

**Authors:** Tisya Banerjee, Elisabetta Moroni, Maximilian Riedl, Giorgio Colombo, Johannes Buchner

**Affiliations:** 1 Center for Protein Assemblies and Department of Bioscience, School of Natural Sciences, Technical University Munich, Garching, Germany; 2 Institute of Chemical Science and Technologies, Italian National Research Council (SCITEC-CNR), Milan, Italy; 3 Department of Chemistry, University of Pavia, Pavia, Italy

**Keywords:** glucocorticoid receptor, integrated stress response, molecular chaperones, phosphorylation, post-translational modification, protein quality control

## Abstract

The molecular chaperone Hsp90 is a major protein folding factor in the cytosol of eukaryotic cells. Its conformational cycle is regulated by various co-chaperones and post-translational modifications (PTMs) such as phosphorylation. Most of the phosphorylation sites are conserved between the 2 isoforms of human Hsp90, Hsp90α and Hsp90β. The analysis of the function of these sites has revealed general functional principles of Hsp90. To what extent isoform-specific phosphorylation regulates Hsp90 function is less well understood. Here, we explore the effect of the phosphorylation of a residue (threonine 446) specific for the constitutionally active isoform Hsp90β. Since T446 is bioinformatically predicted to be phosphorylated by kinases regulating the intrinsic stress response (ISR), this modification links Hsp90 function to the ISR status of the cell in an isotype-specific manner which renders Hsp90 unresponsive to the co-chaperone-mediated modulation of its ATPase activity and consequently affects client maturation. Hsp90β reverts to baseline ATPase-driven chaperoning activity which is no longer intensively regulated by co-chaperones and results in the rewiring of Hsp90-mediated protein quality control.

Hsp90 is a major molecular chaperone in the cytosol of eukaryotic cells. Together with its co-chaperones, it is involved in the maturation and stabilization of hundreds of clients with a wide range of cellular functions (1, 2, 3, 4, 5, 6, 7, 8). Hsp90 consists of 3 different domains. The N-terminal domain (NTD), which bind to ATP (9, 10) is connected to the middle domain (MD), which contains binding sites for clients and co-chaperones as well as residues important for ATP hydrolysis (11, 12, 13), *via* a long charged linker (14, 15, 16). The C-terminal domain (CTD), which mediates dimerization, ends in a disordered tail containing the MEEVD motif which is the binding site for several tetratricopeptide repeat (TPR) domain-containing co-chaperones (17, 18, 19, 20). During the chaperone cycle, Hsp90 undergoes ATP hydrolysis and changes from a “V”-like open state dimerized only at the CTD to a closed conformation, characterized by an additional dimerization of the MD and NTD (11, 12, 21). This conformational transition from the open to the closed state involves intermediates (22) in which a lid closes over the ATP binding site, segments between the 2 NTDs swap and the catalytic loop in the MD binds to the γ-phosphate of ATP (10, 12, 13, 23, 24). These slow structural changes are the rate limiting steps of the cycle (25). Hydrolysis of ATP occurs only after the closed state is reached. This seems to reset Hsp90 (26, 27, 28). The basic cycle is modulated by large number of co-chaperones that bind to Hsp90. Some of them affect specific steps of the cycle or of client processing (12, 29, 30, 31, 32). One of these co-chaperones is the protein Hop which inhibits the ATPase and conformational cycle of Hsp90 and promotes the transfer of client proteins from Hsp70 on to Hsp90 (33, 34, 35, 36). In contrast, Aha1 accelerates the progression of the Hsp90 cycle and, in consequence, stimulates ATP hydrolysis (22, 31, 37, 38). p23 is a co-chaperone that binds to Hsp90 in the closed state and inhibits ATP hydrolysis (12, 22, 39, 40). While these co-chaperones are conserved from yeast to human, there are also co-chaperones which seem to exist only in multi-cellular organisms (8). An example in case is the E3 ubiquitin ligase CHIP which binds to Hsp70 and Hsp90 and targets clients towards degradation (8, 41, 42, 43).

Another layer of regulation is added by a variety of post-translational modifications (PTMs), which have now been notably termed the *Chaperone code.* (29, 44, 45, 46). One of the most prevalent PTMs of Hsp90 is phosphorylation (47). Early on, it was reported that hyperphosphorylation of Hsp90 compromises chaperoning of v-src kinase, a stringent client of Hsp90 (48). Subsequent analysis of individual phosphorylation sites showed that these residues are conformational switches, which are sensitive to changes in the side chain and affect the conformation of distant parts of Hsp90 (29, 30, 46). Examples include T36 and Y38 in the NTD of human Hsp90α (T22 and Y24 in yeast Hsp82, respectively) (49, 50, 51). Phosphorylation of Hsp90 can also dictate client specificity, activation, as well as drug sensitivity (49, 50, 51, 52, 53, 54, 55). In mammals, 2 highly homologous Hsp90 isoforms exist, stress-induced Hsp90α and the constitutionally expressed Hsp90β (56, 57, 58). The 2 isoforms share ∼85% sequence identity (59, 60). While most phosphorylation events studied affect both isoforms, isoform-specific post-translational modifications represent an emerging mechanism by which the 2 major cytosolic Hsp90 paralogs, Hsp90α and Hsp90β, are differentially regulated (61). Hsp90α is uniquely phosphorylated in response to DNA damage (62, 63), to regulate autophagy initiation (64), and to shift co-chaperone binding (65, 66). Hsp90β, in turn, has been reported to be selectively phosphorylated to abolish its ATPase activity (67), with phosphoswitching also driving tumor progression (68).

Although around 20 kinases have been shown to modify Hsp90 (45), the stress-responsive kinases responsible for mediating Hsp90 phosphorylation remain poorly characterized. Also, whether and how the regulation of Hsp90 is linked to the intrinsic stress response (ISR) is largely unknown. Specifically, we are missing information on whether the kinases regulating the intrinsic stress response are also active on Hsp90. Using “The Kinase Library” (PhosphoSitePlus) site (69, 70), we found a phosphorylation site within Hsp90 predicted to be targeted by the major ISR kinases (71, 72, 73, 74). Interestingly, this site, T446 in human Hsp90β, is not found in the stress-induced Hsp90α isoform. This modification had been detected before in phospho-proteomic studies, but the upstream kinase and the effect of the PTM on Hsp90 function remained unknown (51, 75, 76).

To define the consequences of this modification on the function of Hsp90, we analyzed a phosphomimicking mutant and found that modification of this residue makes Hsp90 insensitive to regulation by co-chaperones.

## Results

### Characterization of Hsp90β modified at a phosphorylation site in the middle domain

**Figure 1:**
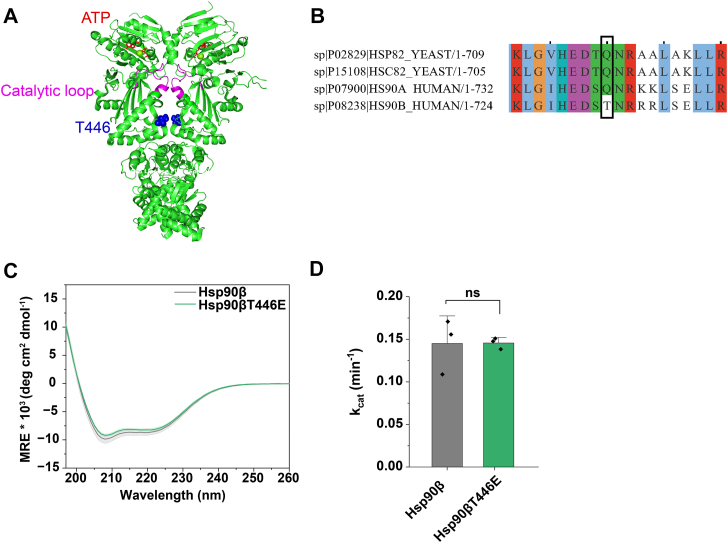
**Hsp90 phosphomimic retains secondary structure and ATPase activity similar to WT**. *A*, the structure of Hsp90β (PDB: 5FWK (104)) with the ATP in *red*, the catalytic loop highlighted in magenta and the T446 residue shown as spheres in *dark blue*. *B*, the sequence similarity of residues between yeast Hsp90 isoforms, the stress inducible Hsp82 and the constitutive Hsc82 with the human Hsp90 isoforms, the stress inducible Hsp90α and the constitutive Hsp90β. The *black rectangle* highlights the corresponding residues in the other Hsp90 isoforms that correspond to T446 in human Hsp90β. *C*, circular dichroism (CD) spectra for Hsp90β compared to the Hsp90βT446E variant. The measurements were performed at a bandwidth of 1 nm in 1 nm steps and 2s per time point. CD measurements have been performed in n = 3 technical replicates. *D*, basal ATPase hydrolysis rates in min^-1^ of Hsp90β and Hsp90βT446E. Data points represent n = 3 technical repeats. Statistical significance was analysed using two-tailed *t* test with Welch correction (ns: not significant, *p* value > 0.05). CD and ATPase assay measurements have been performed in n = 3 technical replicates.

Progress in determining the phospho-proteome landscape in human cells has expanded the repertoire of phosphorylation sites in Hsp90. Among the hits, phosphorylation of the threonine residue at position 446 in the Hsp90β isoform (75, 76) is of particular interest. This residue lies in close proximity to the Hsp90 catalytic loop, which is essential for ATPase activity (Fig. 1*A*). Strikingly, residue T446 is not conserved in human Hsp90α nor in the isoforms of yeast Hsp90 (Fig. 1*B*). Prediction of the kinases involved in its phosphorylation *via* PhosphoSitePlus (69, 70) revealed high scores for the stress kinases. Among the top 10 kinases predicted, there are ISR kinases (77) like HRI, PERK, PKR, and stress-activated kinase EEF2K, amongst several others (Table S1), which were shown previously to partner with Hsp90 in the regulation of the stress response (72, 73, 74, 78).

To define the consequences of the phosphorylation on this residue, we designed a phosphomimicking glutamate mutation at this site (Hsp90βT446E). We compared the secondary structure of the phosphomimic to the wild-type (WT) Hsp90β isoform by far-UV CD spectroscopy and found that the glutamate mutation did not create significant changes in the overall secondary structure of the protein (Fig. 1*C*). The mutation also did not affect the ATPase hydrolysis rates of Hsp90 (Fig. 1*D*). Thus, phosphorylation at this site does not seem to affect the structure and ATP hydrolysis in a general way.

### Phosphomimic of Hsp90 causes a change in the closing kinetics of the Hsp90 dimer

**Figure 2:**
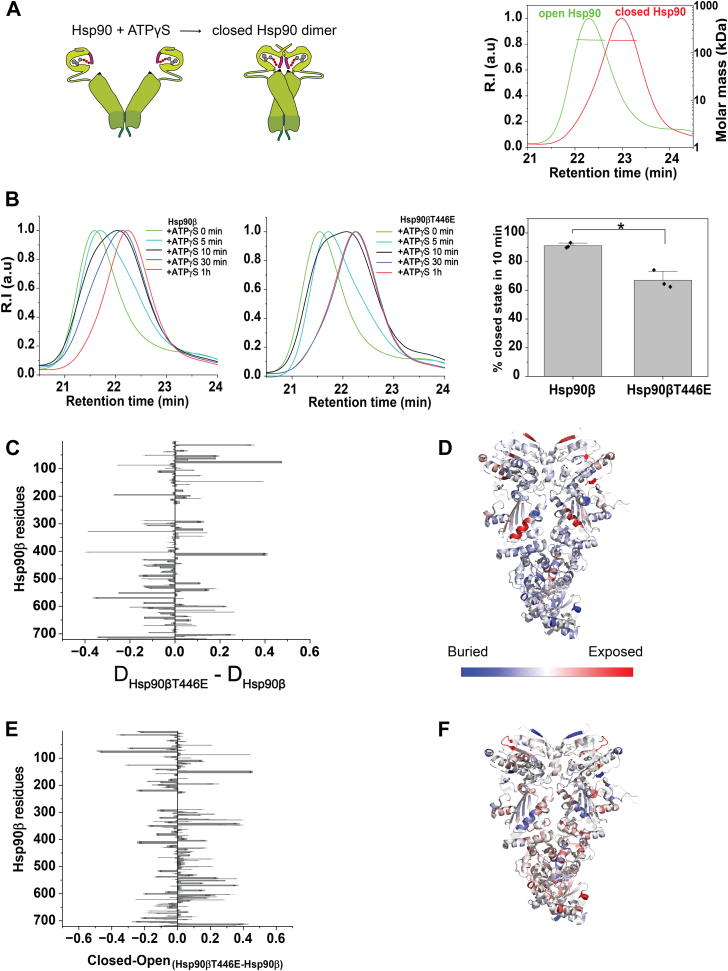
**Hsp90 phosphomimic shows slower closing kinetics due to a different structural conformation**. *A*, the schematic of Hsp90 dimer closing (*left*) and the refractive index peaks observed against retention time in SEC-MALS (*right*) corresponding to their respective molecular mass indicated as *green* and *red lines*. *B*, SEC-MALS experiments were elution profiles showing refractive indices of Hsp90β (*left*) and Hsp90βT446E (*middle*) with ATPγS from 0-1 h time scale. *Right panel*: The closed state percentage for Hsp90β and Hsp90βT446E when incubated with 2 mM ATPγS for 10 min. The percentage of Hsp90 in the closed state is calculated based on the area under the curve obtained from the biGaussian fit on the SEC-MALS Refractive Index profiles. The scatter points are indicative of n = 3 technical repeats. Two-tailed *t* test with Welch correction was used to determine the significance (∗*p* < 0.05). *C*, The difference between the deuterium exchange at the 2 h time point of *apo* Hsp90βT446E and *apo* Hsp90β (D_Hsp90βT446E-_D_Hsp90β_) for the different residues. *D*, the structure of Hsp90β (PDB: 5FWK) highlights the change in relative deuterium exchange at the 2 h time point determined from HDX-MS between Hsp90βT446E and Hsp90β as is indicated in *C*. *E*, the difference between the deuterium exchange at the 2h time point of Hsp90βT446E and Hsp90β for the different residues after transition from open to closed state in the presence of 2 mM ATPγS. *F*, the structure of Hsp90β highlights the change in relative deuterium exchange at the 2h time point determined from HDX-MS between Hsp90βT446E and Hsp90β after the transition from open to closed state, as indicated in *E*. The b values mapped onto the Hsp90 structures and that have been plotted are an average from n = 2 separate experimental repeats. The peptide coverage for all samples has been provided in Fig. S2, *A*–*D*).

Since the PTM could play a role in regulating allosteric conformational changes in the Hsp90 cycle (79), we decided to analyze the closing kinetics of the Hsp90 phosphomimic by analytical SEC, detecting different conformations due to their differences in retention times (80, 81) (Fig. 2*A*). To this end, the N-terminally closed structure of Hsp90 was induced in the presence of the weakly hydrolysable analogue ATPγS (22). We observed that Hsp90α closes faster than Hsp90β (k_closing_ ∼ 0.5 min ^-1^ compared to ∼ 0.2 min^-1^, respectively) (Fig. S1*A*; Table 1). Interestingly, the Hsp90βT446E variant showed the slowest closing kinetics with the dimer staying in an open conformation even after 10 min incubation with ATPγS (k_closing_ of ∼0.1 min^-1^) (Fig. 2*B*; Fig. S1*B*; Table 1).

**Table 1:** Rate of closing of Hsp90 dimer

Hsp90 variant	k_closing_ (min^-1^)
Hsp90α	0.5 ± 0.03
Hsp90β	0.2 ± 0.01
Hsp90βT446E	0.1

Since the phosphomimic at position T446 influences Hsp90 closing but not the ATPase activity, we decided to check its effect on Hsp90 structure in more detail by HDX coupled to mass spectrometry. Comparing Hsp90β with Hsp90βT446E, we observed a pronounced change in the relative deuterium uptake in residues near the ATP binding pocket in the NBD (∼52–79) and in a helix located between the catalytic loop and the phospho-site (403–415) (Fig. 2, *C* and *D*). These regions seem to be less protected in the phosphomimic compared to the WT protein. In contrast, most of the residues in the lower part of the MD, spanning into the CTD, seem to be more buried in the phosphomimic. However, once the dimer is closed with ATPγS (Fig. 2, *E* and *F*), the helix (403–415) shows lower deuterium exchange.

In the presence of ATPγS, more residues in the NBD of Hsp90βT446E were buried compared to Hsp90β. The most notable exception is residues in the NBD (147–154) and in a flexible loop region of the MD upstream of the catalytic loop (340–347), which remain exposed. Thus, the Hsp90βT446E NBD lid and the catalytic loop which are key elements for ATP hydrolysis show conformations similar to that of Hsp90β WT upon closing (Fig. 2*E*), while at the same time stretches of residues in the NBD and MD of the phosphomimic are more exposed compared to the WT isoforms, explaining slower dimer closing kinetics despite similar ATPase rates (Fig. 2*B*, Table 1). Taken together, the T446E mutation results in local and allosteric effects on the conformation of Hsp90β.

### Phosphorylation induces local and global dynamic changes across Hsp90β

**Figure 3:**
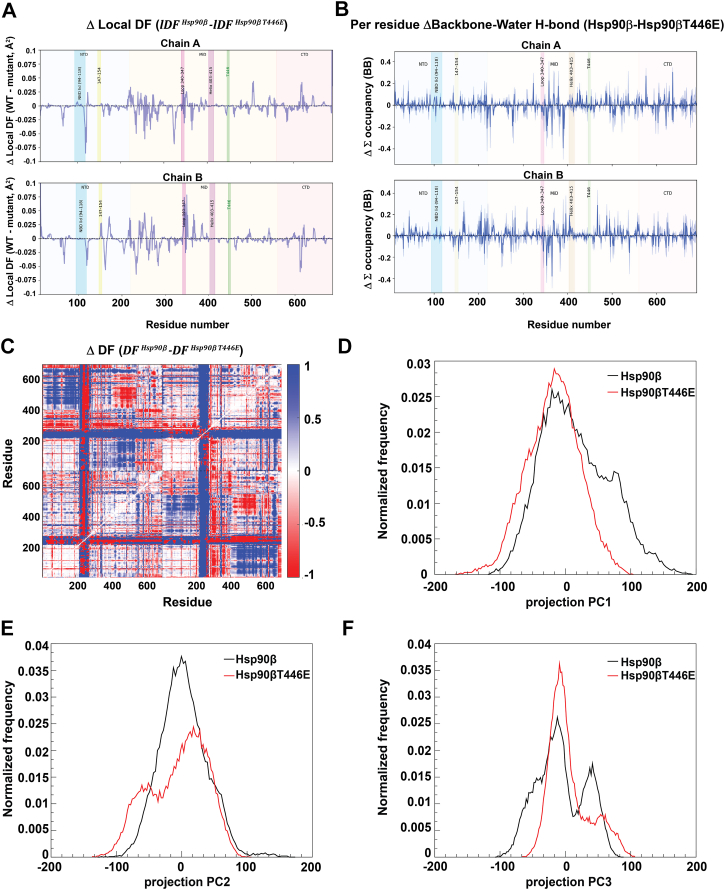
**MD trajectory analysis of Hsp90β WT and T446E**. *A*, per-residue difference in local distance fluctuation, Δ Local DF $\left(l{DF}^{Hsp90\beta }-l{DF}^{Hsp90\beta T446E}\right)$, for chain A and chain B. *B*, per-residue difference in backbone–water hydrogen-bond occupancy for chain A and chain B. *C*, residue–residue distance-fluctuation difference matrix, ΔDF $\left({DF}^{Hsp90\beta }-{DF}^{Hsp90\beta T446E}\right)$; *blue denotes* larger fluctuations in WT, *red denotes* larger fluctuations in T446E, and *white indicates* negligible differences. *D*–*F*, Normalized frequency distributions of the projections of the Hsp90β (*black*) and the mutant Hsp90βT446E (*red*) trajectories onto the first 3 principal components PC1 (D), PC2 (E), and PC3(F).

To further assess the effects of the T446E mutation on the dynamics of the closed ATP-bound conformation of Hsp90β, we performed comparative all-atom Molecular Dynamics simulations of the WT and the mutant variants (82). Local distance fluctuations analysis (Fig. 3*A*), which reports on changes in intrinsic flexibility of contiguous stretches of residues (negative values indicating higher flexibility in the mutant, positive ones indicating increased flexibility in the WT), revealed region-specific effects across all domains. In the NTD, the mutation T446E increased local flexibility in the region adjacent to the ATP-lid and within part of the 16-stranded beta-sheet, while residues 147 to 154 displayed increased rigidity. Overall, NTD local dynamics appeared largely preserved. More pronounced differences emerged in the MD. Within the *β*-sheet region comprising strands 324 to 391 and the catalytic loop, areas of enhanced flexibility alternate with areas of increased rigidity in the mutant. This region is known to mediate communication with the NTD. In the CTD, the mutation induced a prevalent increase in local flexibility, indicating allosteric propagation of its effect.

Analysis of backbone–water hydrogen bond occupancies (Fig. 3*B*) revealed no large differences between WT and T446E across the NTD. Exceptions were residues ∼160 to 180, which were more protected in the mutant, and the helix around residues 40 to 50 as well as the adjacent β-strands (residues ∼70–80), which were more solvent-exposed. Differences were more evident in the MD: the region upstream of the catalytic loop (residues 340–347) was less protected in the mutant, whereas the region downstream (∼350–390) showed increased protection. This region corresponds to part of the β-sheet which mediates communication between the MD and NTD. The CTD displayed a heterogeneous redistribution of water exposure consistent with the observed allosteric effect.

To quantify the effects of the mutation on global dynamics, we calculated residue-residue distant fluctuations (DF), which report on long-range coordinated motions across the whole dimer. Mutation-induced changes were assessed using the DF difference matrix, ΔDF = $\left({DF}^{Hsp90\beta }-{DF}^{Hsp90\beta T446E}\right)$ (Fig. 3*C*), derived from the system specific DF matrices (Fig. S3, *A*–*B*). The results showed that the coupling patterns changed between WT and T446E: the CTD of chain A of the dimer became globally more flexible and decoupled from the rest of its protomer, while the CTD of chain B showed a more heterogeneous pattern with alternating sites of increased rigidity and flexibility. The largest differences emerged in inter-protomer coordination: while the NTD–NTD interactions were largely conserved in the mutant, the MD of chain B became more flexible and lost coordinated motion with chain A. Moreover, inter-protomer NTD–MD interactions appeared more rigidly coupled in the mutant, whereas MD–MD interactions became more decorrelated.

To determine whether the changes in intra- and inter-protomer coordination translate into global collective motions, we performed a principal component analysis (PCA) on the combined trajectories of WT and T446E (Fig. 3, *D*–*F*). This enabled a direct comparison of functional dynamics along the same collective modes. WT and T446E populated partially overlapping but clearly shifted regions of the PC space, indicating that the mutation reshapes the closed-state dynamics. PC1 described a coordinated NTD–MD–CTD rearrangement consistent with the twisting motion associated with closed-state progression. T446E was restricted to a narrower range (Fig. 3*D*). PC2 corresponded to an asymmetric inter-protomer displacement coupling the NTD of one protomer and the CTD of the other. Here, the mutant explores more sub-states than the WT (Fig. 3*E*). PC3, involving relative displacements at the MD-CTD interface implicated in catalytic loop positioning, revealed that T446E is confined to a narrower basin than the WT (Fig. 3*F*). Residue-level contributions to these collective modes are summarized as per-residue RMS fluctuation profiles for PC1-PC3 (Fig. S3, *C*–*E*).

Taken together, MD results showed that T446E does not alter the closed state conformation associated with nucleotide processing but reshapes the balance of collective motions, redistributing flexibility within and between the domains weakening the MD-CTD and inter-protomer coordination, while leaving NTD dynamics predominantly unchanged. This impacts the long-range dynamic allosteric network across the dimer. Interestingly, the regions whose internal dynamics is most affected in T446E are the ones whose role is to recruit co-chaperones and clients.

### Hsp90βT446E abrogates the influence of co-chaperones on Hsp90 ATP hydrolysis

**Figure 4:**
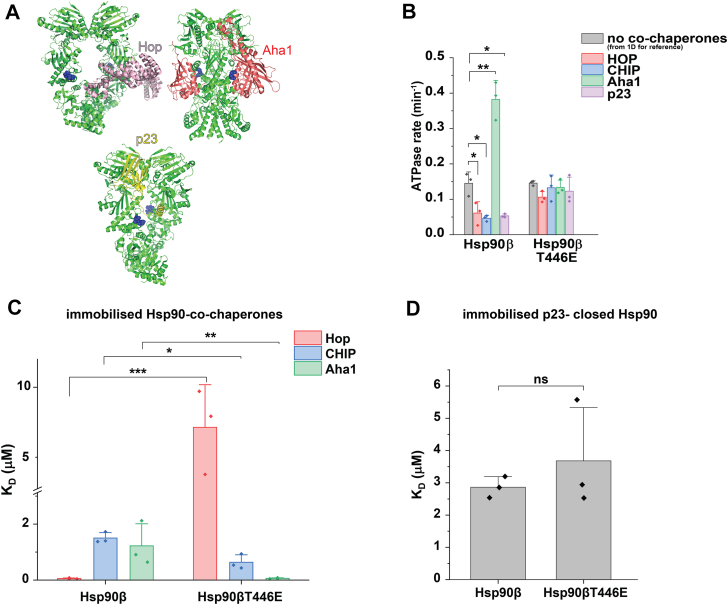
**Co-chaperones lose the ability to modulate Hsp90βT446E ATPase rates but still retain the ability to bind Hsp90**. *A*, the structures of (*left*) Hop bound to Hsp90α (PDB: 7KW7), (*middle*) p23 bound to Hsp90α (PDB: 7KRJ) and (*right*) Aha1 bound to yeast Hsp90 (Hsc82) (PDB: 6XLH). The *blue* spheres indicate the hypothetical position of the phosphosite in the respective Hsp90 isoforms or homolog in yeast. *B*, the ATPase rates in min^-1^ of Hsp90 phosphomimic compared to Hsp90β in absence of any co-chaperone (*black bars* from Fig. 1*D*) and in the presence of co-chaperones Hop (*red*), CHIP (*blue*), Aha1 (*green*) and p23 (*purple*). The diamond-shaped scatter points represent each individual experimental replicate. Statistical significance was determined using two-tailed *t* test with Welch correction (∗∗*p* < 0.01, ∗*p* < 0.05, no mention especially for all samples of Hsp90βT446E: not significant). *C*, the K_D_s obtained from SPR measurements for immobilised Hsp90 phosphomimic and immobilised Hsp90β against Hop (*red*), CHIP (*blue*), Aha1 (*green*). *D*, the K_D_s obtained from SPR measurements of immobilised p23 against Hsp90 phosphomimic and Hsp90β closed with 2 mM ATPγS for 1 h. The diamond scatter points for ATPase and SPR measurements indicate experimental data from n = 3 technical replicates. For *C* and *D* statistical comparison was performed using Welch's *t* test on log_10_ tranformed K_D_ values to stabilize variance across the multi-order magnitude data (∗∗∗*p* < 0.001, ∗∗*p* < 0.01, ∗*p* < 0.05, ns: *p*-value > 0.05). SPR experimental conditions have been explained in detail under Experimental procedures.

Since the Hsp90 MD is the binding site of several co-chaperones (Fig. 4*A*), which influence the Hsp90 ATPase (12, 31, 34, 35, 37, 83, 84, 85), we wondered whether Hsp90βT446E may affect this regulation. For the WT protein, we confirmed the known stimulatory or inhibitory effects of co-chaperones on the Hsp90 ATPase. Surprisingly, however, for Hsp90βT446E, we observed no significant change in ATPase rates in the presence of any of the co-chaperones tested. This is most striking for the ATPase accelerator Aha1 (Fig. 4*B*).

To test whether the effects we observed were due to an altered affinity of the co-chaperones for Hsp90, we monitored binding by surface plasmon resonance (SPR) (Fig. 4, *C* and *D*). For Hop, the affinity for Hsp90βT446E was decreased, whereas Aha1 bound even more strongly to the phosphomimic, and CHIP showed similar affinities compared to WT (Fig. 4*C*; Table S2). Since p23 binds preferentially to closed Hsp90 (86), the affinity of for Hsp90 was checked once dimer closure had been induced with ATPγS (Fig. 4*D*). These experiments showed that p23 bound to the closed phosphomimic with an affinity that was comparable to that of the Hsp90β isoform. Taken together, our results indicate that phosphorylation of T446 does not affect the overall affinity of co-chaperones but interferes with specific functional interactions of Hsp90 with its co-chaperones, which abrogate their regulatory effects on the Hsp90 ATPase.

### Phosphomimic of Hsp90 affects client GR-LBD’s transfer and maturation

**Figure 5:**
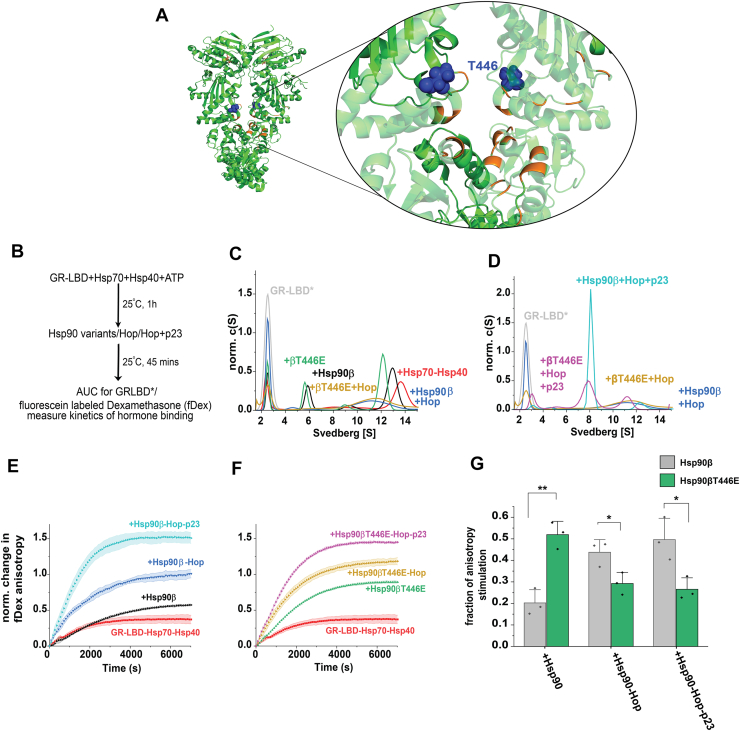
**Hsp90 phosphomimic differentially affects the maturation of the GR-LBD**. *A*, the structure of Hsp90β (PDB: 5FWK) with the phospho-sites depicted as *blue* spheres and the GR-LBD binding sites in *orange*. The GR-LBD binding sites have been obtained from the interactions in corresponding conserved residues highlighted in the Hsp90α in PDB: 7KRJ. *B*, experimental scheme showing the AUC of labeled GR-LBD (GRLBD∗) and hormone-binding assay with labeled Dex (fDex) from Hsp70 complex with different combinations of Hsp90 variants and co-chaperones Hop and p23. *C–D*, AUC data of Atto-488 labeled GR-LBD (GR-LBD∗) in complex with chaperones Hsp70-Hsp40-ATP, Hsp90 variants (Hsp90βT446E has been referred to as βT446E) in the presence of the loading complex comprising co-chaperone Hop *C* and maturation complex comprising of Hop-p23 *D*. In panel *D*, GR-LBD∗ and complexes with Hsp90+Hop from panel *C* have been used to compare the shift in Svedberg values in the presence of p23. *E–F*, hormone binding kinetics of fDex in the presence of chaperone complexes with GR-LBD comprising of Hsp70-Hsp40-ATP-Hsp90-co-chaperones, for WT Hsp90β and phosphomimic Hsp90βT446E. *G*, fraction of stimulation in fDex anisotropy after addition of Hsp90, or Hsp90-Hop or Hsp90-Hop-p23 calculated from the difference between the hormone binding signal obtained in the presence and absence of co-chaperone with Hsp90 from the anisotropy values in *E* and *F*. Data points represent n = 3 technical repeats and Welch’s *t* test has been used to determine significance (∗∗*p* < 0.01 and ∗*p* < 0.05).

Since the phosphorylation site on the MD of Hsp90 is in close proximity to the client binding site for glucocorticoid receptor (GR) (87) (Fig. 5*A*), we wondered whether the chaperoning of GR by Hsp70/Hsp40 is affected in the Hsp90 phosphomimic. We first determined the affinity of Hsp90 for the ligand-binding domain of the glucocorticoid receptor (GR-LBD) by anisotropy (Table S3). We observed similar affinities of ∼ 1.5 μM for the T446E phosphomimic as well as WT Hsp90β. We then performed AUC experiments with labeled GR-LBD (GR-LBD∗) to check for complex formation with Hsp70/Hsp40 and Hsp90 (Fig. 5, *B*–*D*). As reported previously (36), in the presence of Hsp70-Hsp40-ATP and GR-LBD∗ large complexes at ∼ 16 S are observed. Upon addition of Hsp90, the GR-LBD∗-Hsp90β complexes sedimented at lower Svedberg values compared to WT protein (Fig. 5*C*), thus indicating differences in complex conformation. When Hop was added to test the ability of Hsp90 phosphomimic to form loading complexes with Hsp70, we observe similar complexes at ∼ 11 S for both WT and phosphomimic Hsp90 that indicating similar loading complex formation, with Hop allowing for the dissociation of larger Hsp70-GR complexes (36). However, there is a higher intensity of free GR-LBD∗ at ∼ 2.5 S released from the WT Hsp90 loading complex. Furthermore, we checked for the ability of the phosphomimic to form GR-LBD maturation complex with p23 (Fig. 5*D*). Here, we observed that GR-LBD readily forms complexes comprising of WT Hsp90β and p23 with high intensity at ∼ 8 S. The phosphomimic also shows similar complexes, but at the same time retains the complex with Hsp70-Hsp90-Hop, as is indicated by the broader peak at ∼ 11 S. Thus, Hsp90 phosphomimic forms complexes with the client GR and co-chaperones that appear to sediment differently from WT, thereby suggesting a change in the nature of the Hsp90 cycle.

To test whether the GR-LBD reached the native state in the maturation complexes in the presence of the T446E variant, we performed hormone binding experiments using fluorescently labeled dexamethasone (fDex) (Fig. 5, *B*, *E*–*G*). Conforming with previous reports, GR-LBD in the presence of Hsp70-Hsp40 and ATP is partially unfolded and loses the ability to bind to fDex (88, 89, 90). However, in the presence of Hsp90, and co-chaperones like Hop that allow client transfer and p23 that allows client maturation, the GR-LBD refolds which is reflected in an increase in the fDex binding kinetics as monitored by anisotropy (36) (Fig. 5*E*). For the phosphomimic, there is an initial increase in the anisotropy signal compared to the WT protein in the absence of any co-chaperones, indicating, the GR follows a different client processing cycle compared to WT Hsp90 which could be due to the differing interaction between GR and phosphomimic as has been suggested through our AUC experiments (Fig. 5*C*) and MD simulations. However, after each step of co-chaperone addition, the step-wise increase of fDex binding is higher for the WT protein compared to the phosphomimic (Fig. 5, *F* and *G*). This implies that the co-chaperone-mediated processing of the GR-LBD is compromised in the Hsp90βT446E phosphomimic.

## Discussion

Since Hsp90 is of key importance for the conformational regulation of many cell-fate-determining proteins under physiological conditions, its function needs to be tightly regulated. This is achieved by a large set of post-translational modifications, most prominently phosphorylation. The presence of cognate and stress-regulated isoforms allows for further adjusted activities by specific phosphorylation events (61, 62, 91, 92). A major gap in our understanding in this context is whether and how Hsp90 is regulated by the kinases of the ISR. One could envision different scenarios: Hsp90 could be left unregulated by the ISR. This would maintain the same chaperoning capacity even under conditions of reduced protein synthesis. Thus, on the one hand, the Hsp90 clients, which include many transcription factors and protein kinases, would still be chaperoned to the active state implying that the stress state of the cell does not affect their maturation. This may be detrimental for the cell. On the other hand, there would be additional chaperone power available as the number of newly synthesized proteins is decreased. In a second scenario, which our results support, the ISR involves the downregulation of Hsp90 activity *via* phosphorylation. Thus, Hsp90β activity must have negative consequences under stress conditions (93). Unfortunately, a detailed characterization of the 2 human Hsp90 isoforms and their differences is still missing. The differences in expression of the 2 isoforms suggest that these exist: Human Hsp90β is 2.5 times more abundant under physiological conditions than Hsp90α, while the Hsp90α isoform becomes 7-fold more abundant under heat stress (58). For the yeast Hsp90s we know that the stress-induced isoform Hsp82 has higher stability, enhanced refolding capacity, and a slight preference for clients with higher isoelectric points (94, 95, 96). However, 97% of the potential phospho-sites are conserved across Hsp82 and Hsc82 (51), making the comparison only partially informative, as here this isoform-specific phosphorylation is missing.

For the human isoforms, the ISR kinase predicted bioinformatically seems to specifically modulate the function of the constitutive Hsp90β isoform leaving the stress-induced Hsp90α isoform untouched as the respective threonine residue in position 446 is absent there. The Hsp90β regulation we identified is both broad and specific: the glutamate variant, that carries a side chain negative charge and sterically acts as a phosphomimic (97), negates the effects of several co-chaperones that influence the Hsp90 ATPase, seemingly irrespective of their binding sites on Hsp90 and whether they act in the cycle at early stages (like Hop and CHIP) or in later parts (like p23). Interestingly, our analysis showed that binding itself is not compromised in the phosphovariant. For Aha1 we even observed an increase in the affinity for the Hsp90 phosphomimic. The discrepancy between the increased affinity of Aha1 and the absence of Hsp90 ATPase modulation likely arises from differences in local dynamics, as revealed by our MD simulations, which show increased flexibility in the middle-domain β-strands interspersed with transient rigid regions, together with greater water exposure upstream of the catalytic loop and increased residue protection downstream. This points towards a flexibility of the middle domain to accommodate Aha1 binding without promoting the conformational changes required for ATPase acceleration. The new regulatory concept revealed here, is that phosphorylation seems to disrupt the allosteric network of Hsp90 connecting ATP hydrolysis and client processing. Thus, while the co-chaperones still bind they do not affect Hsp90 function. This profile and the predicted connection to the ISR kinases sets it apart from phosphorylation-induced changes in Hsp90 function reported before in which the affinity of a specific co-chaperone was affected (61, 98, 99).

Our analysis suggests that the basis for the functional alterations observed is conformational changes mainly in the Hsp90 MD. HDX-MS suggests that in Hsp90βT446E structural elements in the MD result in an altered conformation that shifts when dimer closing was induced with ATPγS. The molecular dynamics simulation data are also in alignment with these data, suggesting allosteric changes in the MD and CTD of the Hsp90 variant, whereas the dynamics in major regions of the NTD remain unchanged compared to the WT protein. The simulations also suggest that allosteric changes in the internal dynamics of Hsp90 sub-structures are important for client and co-chaperone recognition, consistent with the experimentally observed changes in affinities for co-chaperones and perturbations of client maturation. The results further highlight the concept of an allosteric flow of “conformational information” through Hsp90 during the chaperone cycle, which had been established in the context of the discovery of conformational switch points in different domains of Hsp90 (29, 30, 80).

**Figure 6:**
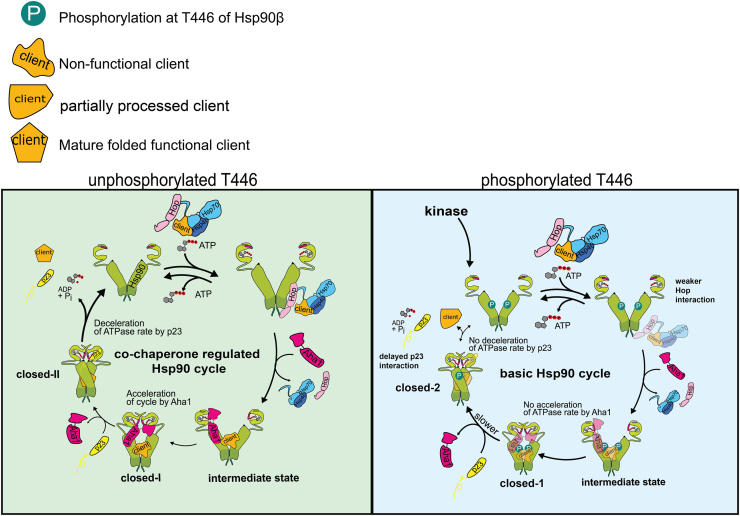
**Model of the basic chaperone cycle of the phosphorylated Hsp90β**. Hsp90β under non phosphorylated conditions (*left panel*) undergo the usual chaperoning cycle induced by co-chaperones – client transfer mediated by Hop, Aha1 stimulated ATP hydrolysis and p23 mediated final client maturation when Hsp90 is in the closed-2 state. Kinase–mediated phosphorylation of Hsp90β at T446 (*right panel*) slows nucleotide-induced conformational closing, leading to accumulation of a closed-1–like state. Although this conformation would normally facilitate Aha1-driven transition to the closed-2 state, this effect is impaired in the T446 mutant. In addition, phosphorylation delays and disrupts p23-mediated stabilization of the closed-2 state and its ability to decelerate ATP hydrolysis. As a result, co-chaperone-dependent client maturation is compromised, while basal Hsp90β activity is retained. The reduced transparency in co-chaperones and client in the right panel is representative of disruption in cycle modulation and client processing.

In summary, our proposed model depicts the consequences of phosphorylation of T446 of Hsp90β on the regulation of the Hsp90 chaperone cycle (Fig. 6). It slows the nucleotide-induced closing, populating a conformation which might resemble the closed-1 state (11, 13, 31, 85, 99, 100). This should favor the function of Aha1 to induce the closed-2 state. However, this effect is blocked in the mutant. Also, the co-chaperone p23, which stabilizes the closed-2 state (22, 39, 86), loses its ability to decelerate ATP hydrolysis. In combination, these differences seem to compromise client maturation. Thus, T446 phosphorylated Hsp90β maintains its basal activity but co-chaperone-mediated processing of clients like transcription factors, E3 ligases, and kinases (101) is decreased—which adds an attenuation of protein folding to the attenuation of translation activity mediated *via* the ISR signaling pathway (77). While our study focuses on ISR kinase-mediated phosphorylation of Hsp90 as a proposed mechanism for disrupting co-chaperone regulation of its ATPase cycle, kinases may also target co-chaperones directly, as shown for Cdc37, where cycling between distinct Yes- or CK2-mediated phosphorylation states regulates kinase client activation (102) and Aha1 phosphorylation at Y223 by c-Abl enhancing Hsp90 ATPase activity and rewiring client chaperoning (103). Disentangling the relative contributions of direct Hsp90 phosphorylation *versus* co-chaperone phosphorylation by kinases connected to ISR signaling, therefore, remains an important goal for future work. Our study aimed to lay the groundwork for characterizing this isoform-specific Hsp90 phosphorylation site, opening the possibility of future exploration *in vivo* or in cells to investigate the connection between the *Chaperone code* and the predicted stress kinases.

Moreover, these observations are specific for the constitutively expressed isoform of Hsp90, which represents the majority of the Hsp90 cellular population. Modifying this phosphorylation site creates widespread changes in the allosteric network across the Hsp90 structure with increased dynamics in co-chaperone and client binding regions that lead to rewiring of the chaperoning process from co-chaperone regulation. This effect selectively alters the constitutive “household” functions of Hsp90 while leaving the stress-related functions of Hsp90α untouched. Defining the underlying differences between the 2 isoforms will help to further understand the connection between chaperone function and the ISR.

## Experimental procedures

### Kinase prediction for Hsp90 phosphorylation site

The kinase prediction for T446 phosphorylation in Hsp90β was performed using the Kinase Library website (69, 70) with the tool Score Site as per the instructions mentioned in the website. The primary sequence of Hsp90β was used as input, with the phosphorylation site of interest containing this amino acid in lowercase, followed by the “∗” symbol. The prediction tool then returns the kinases that are top hits, assigning a score based on position-specific scoring matrices (PSSM) that match Hsp90 amino acids to sequences preferred by kinases. From the list generated by the software, we shortlisted the first 10 kinases based on their score rank in Table S1.

### Mutagenesis, protein expression and Purification

Single amino acid point mutation in human Hsp90βT446E was introduced utilising the Q5 site-directed mutagenesis kit (New England Biolabs) as per the manufacturer’s instructions. The successful introduction of the mutation was confirmed through Sanger sequencing (Eurofins).

Human Hsp90 isoforms and variants – Hsp90α, Hsp90β and Hsp90βT446E - containing a 6X-His tag were expressed in *Escherichia coli* with 1 mM IPTG at 37 °C for 4 h. The cells were harvested and resuspended in Ni-NTA Buffer A (50 mM Na_2_HPO_4_, 300 mM NaCl, 10 mM Imidazole, pH 7.8) with protease inhibitor HP (Serva electrophoresis GmbH) and DNase I (Roche). The cells were lysed using a French Press (Constant Systems Limited) at 1.8 kbar and 8 °C and then centrifuged at 48,384 g (Beckman Avanti J-26 XP, Beckman Coulter) for 1 h at 4 °C. The supernatant containing the lysate was loaded onto a 5 ml His-Trap FF (5 ml FF, GE Healthcare) pre-equilibrated with Ni-NTA Buffer A. Elution was performed in 60% Ni-NTA Buffer B (Ni-NTA Buffer A with 500 mM Imidazole). The eluted fractions were pooled and diluted in Resource Q Buffer A (40 mM HEPES pH 7.5, 20 mM KCl, 1 mM EDTA, 1 mM DTT). Elution was performed in a Resource Q column (6 ml, GE Healthcare) over a gradient of 0 to 50% Resource Q Buffer B (40 mM HEPES pH 7.5, 1 M KCl, 1 mM EDTA, 1 mM DTT). The peak fractions were run on SDS PAGE, and the purest fractions were collected and concentrated together to 5 ml and loaded onto a Superdex 200 pg HiLoad 16/60 column (GE Healthcare) pre-equilibriated with Size Exclusion (SEC) Buffer (40 mM HEPES pH = 7.5, 150 mM KCl, 5 mM MgCl_2_, 1 mM DTT). The peak fractions were run on an SDS-PAGE gel, and the purest fractions were pooled, flash frozen in liquid nitrogen, and stored at −80 °C for further use.

Human Hsp70 and yeast Ydj1 were expressed from the pET28b vector with a cleavable 6X His-tag sequence recognizable to SUMO protease. The cells expressing Hsp70 and Ydj1 were grown to an O.D of 0.6, followed by 1 mM IPTG induction at 30 °C overnight and 4 h, respectively. The cells were harvested and resuspended in Ni-NTA buffer A (40 mM HEPES pH 7.5, 150 mM KCl, 350 mM NaCl, 20 mM MgCl_2_, 5% Glycerin, 10 mM Imidazole, 2 mM β-mercaptoethanol for Hsp70 and 40 mM NaH_2_PO_4_, 500 mM NaCl, 20 mM Imidazole, 2 mM β-mercaptoethanol, 10% glycerin for Ydj1). The supernatant, after cell lysis, was loaded onto the 5 ml His-Trap FF column pre-equilibrated with Ni-NTA Buffer A + 0.5 mM ATP. The column was washed with Ni-NTA Buffer A containing ATP. The protein was eluted at 60% Ni-NTA Buffer B (Ni-NTA Buffer A with 500 mM Imidazole). The eluted fractions were pooled and diluted in 1:5 in cold water, SUMO protease was added. Dialysis was carried out against cold Ni-NTA Buffer A overnight. The cleaved protein was then purified from the His-tagged SUMO protease and undigested protein by another Ni-NTA chromatography, where the flow-through containing the cleaved protein was collected. These fractions were pooled and concentrated to 5 ml and loaded onto a Superdex 200 pg 26/60 column for Hsp70 and Superdex 75 pg for Ydj1. The purest fractions as observed in an SDS-PAGE gel were pooled, concentrated and stored similar to Hsp90 mentioned above.

Human Hop was purified without any tag in *E. coli*. The cell lysate was loaded onto a Q Sepharose ion exchange (IEX) column pre-equilibrated with IEX Buffer A (20 mM HEPES, 5 mM MgCl_2_, pH 7.6). Elution was performed over a linear gradient of IEX Buffer B (IEX Buffer A + 500 mM KCl) over 12 column volumes. The purest fractions from SDS-PAGE gel were pooled and dialyzed against 10 mM potassium phosphate buffer, pH 7.5. The dialyzed protein was then loaded onto a hydroxyapatite column (HAT) pre-equilibrated in the dialysis buffer and eluted over a linear gradient of 0 to 400 mM potassium phosphate buffer, pH 7.5. The purest fractions obtained from an SDS-PAGE gel were pooled and concentrated to 10 ml and loaded on a 16/60 Superdex 75 pg column pre-equilibrated in 25 mM HEPES pH 7.5, 150 mM KCl, 5 mM MgCl_2_, and 2 mM DTT. The pure fractions from an SDS-PAGE gel were pooled and collected as mentioned for Hsp90.

The human CHIP was expressed with a TEV protease-cleavable 6x-His tag in *E. coli* with 1 mM IPTG at 37 °C for 4 h. The cells were harvested and resuspended in Ni-NTA Buffer A (50 mM Na_2_HPO_4_, 300 mM NaCl, 10 mM Imidazole, pH 7.8) with protease inhibitor HP and DNase I. The cells were lysed using a French Press at 1.8 kbar and 8 °C and then centrifuged at 48,384 g for 1 h at 4 °C. The supernatant containing the lysate was loaded onto a 5 ml His-Trap FF pre-equilibrated with Ni-NTA Buffer A. Elution was performed in 60% Ni-NTA Buffer B (Ni-NTA Buffer A with 500 mM Imidazole). The eluted fractions were pooled and diluted in 1:5 cold water, TEV-protease was added. Dialysis was carried out against cold Ni-NTA Buffer A overnight. The cleaved protein was then purified from the His-tagged TEV protease and uncut protein by another Ni-NTA chromatography, where the flow-through containing the cleaved protein was collected. These fractions were pooled, concentrated to 5 ml, and loaded onto a Superdex 75 pg 26/60 column. As observed in an SDS-PAGE gel, the purest fractions were pooled, concentrated, and stored similarly to Hsp90 mentioned above.

Human GR-LBD (aa 527–777, F602S/A605 V/V702 A/E705 G/M752 T) is a stable construct with a TEV-protease cleavable 6X-His tag that was expressed in ZYM 5052 media supplied with 250 μM Dexamethasone (DEX) at 18 °C overnight. The cells were harvested and washed with ice-cold PBS. Cells were resuspended Ni-NTA Buffer A Buffer (50 mM Tris, 100 mM NaCl, 10 mM Imidazole, 2 M Urea, 5 mM MgCl_2_, 2 mM β-mercaptoethanol, 50 μM DEX, pH 7.9), DNase I and protease inhibitor HP. The cells, after lysis, were loaded onto a 5 ml His-trap FF column equilibrated with Ni-NTA Buffer A + 10% Glycerin. The column was washed in a gradient of Buffer A followed by elution in Ni-NTA Buffer B (Ni-NTA Buffer A + 350 μM Imidazole). Overnight dialysis was performed for the pooled eluted fractions with TEV protease against cold dialysis buffer (50 mM Tris, 100 mM NaCl, 2 mM β-mercaptoethanol, 10% Glycerol, 0.5% CHAPS, 50 μM DEX, pH 7.9). The cleaved protein was then purified from the His-tagged TEV protease and uncut protein by another Ni-NTA chromatography, where the flow through containing the cleaved protein was collected. These fractions were pooled, concentrated to 5 ml, and loaded onto a Superdex 75 pg 16/60 column. The purest fractions, as observed in an SDS-PAGE gel, were pooled, concentrated, and stored similarly to Hsp90 mentioned above.

p23 was provided by Dr Jannis Lawatscheck and Aha1, was obtained from Dr Vinay Dahiya.

### Far UV Circular dichroism measurements

For all Hsp90 constructs, Far UV CD spectra were measured using a Chirascan-plus CD spectrometer (Applied Photophysics, Leatherhead, England) between 197 nm and 260 nm. The samples were prepared at a concentration of 3 μM in a 0.5 mm quartz cuvette (Hellma Analytics, Müllheim, Germany) and the measurements were performed at a bandwidth of 1 nm in 1 nm steps and 2s time per point. The spectra show an average of three independent measurements. The data were plotted using OriginPro (OriginLab).

### ATPase assay

The ATPase activity for the different constructs of Hsp90 was measured using a regenerative ATPase assay as described before (40). NADH reduction is detected by a decrease in the absorbance at wavelength 340 nm. 5 μM of different constructs of dimer Hsp90 was incubated with or without 20 μM co-chaperone in 40 mM HEPES pH 7.5, 150 mM KCl, 5 mM MgCl_2_ at 37 °C and ATPase premix buffer [NADH, Phosphoenol pyruvate, pyruvate kinase, and Lactate dehydrogenase (Sigma-Aldrich)] for 30 min. The baseline signal was checked at 340 nm on the Infinite M Nano plus plate-reader (Tecan) for 10 min, followed by the addition of 2 mM ATP and the absorbance was measured further for 20 min. Finally, 50 μM of Radicicol (Carl Roth) was added to inhibit Hsp90 ATPase activity, and the absorbance was measured for another 10 min. The background ATPase signal obtained after addition of radicicol is subtracted from the signal that was obtained when ATP was added. ATPase activity was calculated from the absorbance-vs-time slopes following Beer-Lambert’s law using the NADH molar extinction coefficient (6200 M^-1^ cm^-1^).

### Size exclusion chromatography with multi-angle light scattering (SEC-MALS)

SEC-MALS measurements were performed as described before (80) with slight modifications. 5 μM Hsp90 was closed with the poorly hydrolysable form of ATP (ATPγS) in 40 mM HEPES (pH 7.5), 150 mM KCl, 5 mM MgCl_2_ and 1 mM DTT, and incubated as per experimental time points. 100 μl of the sample was loaded onto a Superdex 200 pg Increase 10/300 Gl column pre-equilibriated with the closing buffer and connected to an HPLC System (Shimadzu), and elution profiles of Hsp90 at different closing states based on its hydrodynamic radius were determined by a Dawn Heleos multi-angle light scattering detector (Wyatt Technology). The Astra 5.3.4 software (Wyatt Technology) was used for data analysis. Refractive index elution profiles were fitted onto a Bi-Gaussian fitting function in OriginPro to analyse the fraction of Hsp90 that was closed.

### HDX-MS measurements

HDX-MS was carried out on a system consisting of a Synapt G2-Si MS, ACQUITY M-Class UPLC, HDX Manager and a LEAP HDX-2 automation platform from Waters as described before (105). 30 μM of initial protein was diluted in a 1:20 ratio in 40 mM HEPES, 150 mM KCl, 5 mM MgCl2 pH 7.5 and 1 mM DTT with D2O. At 0 s, the experiment is carried out without deuterium. After 10, 60, 600, 1800 and 7200 s the Deuterium exchange is stopped with a quenching solution comprising of 200 mM Na2HPO4, 200 mM KH2PO4, 4 M GdmCl, pH 2.3 in a 1:1 ratio at 1 °C. The samples were transferred onto a Pepsin column (Waters Enzymate BEH Pepsin Column 2.1 mm × 30 mm), and the pepsin digestion took place at 0 °C. ACQUITY UPLC BEH C18 1.7 μM VanGuard Pre-Column 2. 1 × 5 mm was used to separate the peptides, and then they were loaded onto an analytical column (ACQUITY UPLC BEH C18 1.7 μM 1.0 × 100 mm) before the peptides were measured by MS. The HDX data were analyzed using the DynamX software (Waters). The b values obtained give the deuterium exchange, which can be compared between mutants or different Hsp90 conformations for every residue and plotted as a butterfly plot in OriginPro and mapped onto the protein structures in PyMOL.

### System preparation for molecular dynamics simulations

The molecular systems were prepared starting from the crystal structures of the human Hsp90β in complex with an ATP analog (PDB ID 8EOB). Missing residues were modeled using MODELLER (106), and the best model was selected according to the Discrete Optimized Protein Energy (DOPE) score (107, 108, 109). Two systems were prepared (1): the wild-type Hsp90β and (2) the mutant T446E. The point mutation was introduced with the *Prime MM-GBSA residue scanning workflow* (Schrödinger Suite), which builds the desired amino-acid substitution and locally optimizes side chains of residues close to the mutation by constrained energy minimization. The ATP molecule was docked with Glide (Schrödinger LLC, Schrödinger Release 2025–2) in the nucleotide binding sites of each protomer, taking into account the position of the co-crystalized ANP molecule, and preserving the coordination geometry of the Mg2+ ions. The *tleap* utility in the *AmberTools* suite (version 25) (110) was used to add hydrogens, with the protonation states of titratable side chains of Asp, Glu, Arg, Lys, Tyr, His and Cys residues predicted using the H++ program at pH 7.0 (111). Each system was solvated in a truncated octahedral box with *OPC* water molecules (112), ensuring a minimum distance of 15 Å between the protein and the box edges, and neutralized by adding the appropriate number of Na^+^ or Cl^-^ counterions ions modeled using the parameters developed by Li/Merz (113). Additional Na^+^ and Cl^-^ ions were randomly placed in the solvent to reach a final salt concentration of 150 mM, in order to mimic physiological ionic strength.

### Molecular dynamics simulations

Molecular dynamics simulations were performed with the AMBER program (version 24) (D.A. Case, H. M. Aktulga, K. Belfon, I. Y. Ben-Shalom, J. T. Berryman, S. R. Brozell, F. S. Carvahol, D.S. Cerutti, T.E. Cheatham, III, G. A. Cisneros, V. W. D. Cruzeiro, T. A. Darden, N. Forouzesh, M. Ghazimirsaeed, G. Giambaşu, T. Giese, M. K. Gilson, H. Gohlke, A. W. Goetz, J. Harris, Z. Huang, S. Izadi, S. A. Izmailov, K. Kasavajhala, M. C. Kaymak, I. Kolossv∖'a ry, A. Kovalenko, T. Kurtzman, T. S. Lee, P. Li, Z. Li, C. Lin, J. Liu, T. Luchko, R. Luo, M. Machado, M. Manathunga, K. M. Merz, Y. Miao, O. Mikhailovskii, G. Monard, H. Nguyen, K. A. O'Hearn, A. Onufriev, F. Pan, S. Pantano, A. Rahnamoun, D. R. Roe, A. Roitberg, C. Sagui, S. Schott-Verdugo, A. Shajan, J. Shen, C. L. Simmerling, N. R. Skrynnikov, J. Smith, J. Swails, R. C. Walker, J. Wang, J. Wang, X. Wu, Y. Wu, Y. Xiong, Y. Xue, D. M. York, C. Zhao, Q. Zhu, and P. A. Kollman (2025), Amber 2025, University of California, San Francisco) (www.ambermd.org) using the ff19SB protein force field (114), the Li/Merz parameters for Mg2+ (115). System preparation (minimization and initial equilibration) used the *sander* utility, whereas production runs were carried out with *pmemd.cuda*. Three independent replicas were generated for each system by assigning different initial velocity seeds, each comprising a short minimization/equilibration phase (total 10.069 ns) followed by a 750 ns constant Number of particles (N), Pressure (P) and Temperature (T), NPT, production run.

After solvation, 2 consecutive energy minimizations (300 steps each; 150 steepest descent + 150 conjugate gradient) were performed with an 8.0 Å non-bonded cutoff, first with positional restraints of 5 kcal mol^-1^ Å^-2^ on all heavy atoms and then without restraints. The minimized systems were equilibrated in the constant Number of particles (N), Volume (P) and Temperature (T), NVT, ensemble using a 9 ps simulated annealing protocol, with all solute atoms restrained by a 10 kcal mol^-1^ Å^-2^ potential to relax only the solvent: the system was gradually heated from 25 K to 400 K (0–3 ps), then the temperature was maintained at 400 K to allow for solvent relaxation (3–6 ps), finally in the cooling phase (6–9 ps) the system was slowly cooled from 400 K to 25 K, facilitating optimal solvent packing around the solute. Temperature was controlled with a Berendsen thermostat (116) using tight coupling (τ = 0.2 ps) during heating/plateau and looser coupling (τ from 2.0 to 1.0 ps) during cooling.

The temperature was then increased from 25 K to 300 K over 20 ps of NVT dynamics with periodic boundary conditions, an 8.0 Å cutoff, and positional restraints of 5 kcal mol^-1^ Å^-2^ on C_α_ atoms to preserve the overall fold. A Langevin thermostat (117) (collision frequency 0.75 ps^-1^) was used for temperature control, and bonds involving hydrogen atoms were constrained with SHAKE (118), allowing a 2 fs time step.

Subsequently, systems were equilibrated for 10.04 ns in the NPT ensemble at 300 K and 1 bar, with periodic boundary conditions, an 8.0 Å cutoff, and SHAKE, constraints on bonds to hydrogen. Pressure was maintained with a Berendsen barostat (relaxation time 1.0 ps) and temperature with a Langevin thermostat (collision frequency 1.0 ps^-1^); positional restraints on C_α_ atoms were gradually reduced from 3.75 to 1.75 kcal mol^-1^ Å^-2^ over the first 40 ps and then removed for the remaining 10 ns. Production MD was finally run in the NPT ensemble at 300 K and 1 bar using a 2 fs time step, Langevin thermostat (collision frequency 1.0 ps^-1^), and Berendsen barostat (relaxation time 1.0 ps). Short-range non-bonded interactions employed an 8.0 Å cutoff, with long-range electrostatics treated by the particle mesh Ewald method (119); SETTLE was used for water and SHAKE for all other bonds involving hydrogens.

Trajectories were processed and analyzed with cpptraj (AmberTools, version 25) and in-house Python scripts.

Distance fluctuation (DF) (120, 121) analysis was performed separately for the wild-type protein (WT) and the mutant protein (T446E) using trajectories obtained by concatenating the 3 independent replicas of each system. For each system, DF values were computed for all residue pairs i, j as $D_{ij}=\left\langle {\left(d_{ij}-\left\langle d_{ij}\right\rangle \right)}^{2}\right\rangle$ where d_ij_ is the time-dependent distance between the C_α_ atoms of residues i and j, and angular brackets denote the time average over the full trajectory. This yields an N x N DF matrix describing the variance of the relative distance for all residue pairs. Mutation-induced changes were characterized by the difference matrix ${\Delta D}_{ij}=D_{ij}^{WT}-D_{ij}^{T446E}$.

A local distance fluctuation (local DF) profile was derived as a one-dimensional sequence descriptor from the DF matrix. For each residue i, local DF was calculated as ${lDF}_{i}=\frac{1}{4}\left(D_{i,i-2}+D_{i,i-1}+D_{i,i+1,}+D_{i,i+2}\right)$, excluding residues within 2 positions from the termini. Profiles were computed separately for WT and T446E and compared by subtraction $\left(l{DF}^{WT}-l{DF}^{T446E}\right)$ to highlight mutation-induced changes in local backbone flexibility.

Backbone–water hydrogen bonds were identified using the *hbond* command in cpptraj, using the default geometric criteria (donor–acceptor distance 3.5 Å; H–D–A angle 135°), with backbone amide N–H groups as donors and solvent as acceptor.

For each trajectory, hydrogen-bond occupancies (fraction of frames that satisfy the geometric criteria) were used to build per-residue profiles, which were averaged over replicas. Uncertainties were estimated as the standard error of the mean.

Principal component analysis (PCA, essential dynamics) was performed on concatenated WT and T446E trajectories, after alignment of all frames to a common reference to remove overall roto-translational motion, restricting the analysis to backbone atoms and excluding the highly flexible loop 220 to 277 in both protomers. The analysis focused on PC1–PC3, which together account for 64% of the total variance. The concatenated trajectories were projected onto these modes to obtain the corresponding conformational distributions for each system.

Mode-specific fluctuation profiles were obtained by computing per-atom RMS fluctuations associated with each principal component using the corresponding eigenvectors, thus identifying residues that contribute most strongly to each dominant motion.

### Surface plasmon resonance (SPR)

Surface Plasmon Resonance data was collected on a Biacore X100 (Cytiva) as mentioned previously (122) with the following modifications. A CM5 chip was used to covalently link the different variants of Hsp90 or co-chaperone p23 *via* amine-coupling as per manufacturer’s instructions. About 500 resonance units of Hsp90 and around 100 units of p23 were coupled to flow cell two of the chip, and flow cell one was blocked and activated to obtain similar surfaces. A concentration gradient of the co-chaperones Aha1, Hop or CHIP (5 nM – 20 μM) was used to measure their binding affinity from immobilized Hsp90 mutants. Similarly, a concentration gradient of different Hsp90 variants (between 30 nM – 20 μM) (pre-incubated for an hour with 2 mM ATPγS) was applied to measure their affinity against immobilized p23 on the CM5 chip. The Biacore Evaluation Software (Cytiva) was used to calculate the K_D_ values, based on the Langmuir binding model.

### Protein labeling

The GR-LBD was made to undergo extensive dialysis in dialysis buffer (20 mM HEPES, 150 mM NaCl, 0.04% CHAPS pH 7.5) at 4 °C to remove any dexamethasone and DTT. The Atto-488 maleimide dye (ATTO-TEC) was then added in a 3-fold excess to *apo* GR and the labeling reaction was carried out at room temperature in the dark for 1.5 h. The labeling reaction was stopped with 5 mM DTT and then the free dye is removed from the labeled GRLBD by extensive dialysis in the same dialysis buffer mentioned earlier with 2 mM DTT in 4 °C. The degree of labeling was calculated according to the manufacturer’s instructions.

### Fluorescence-anisotropy-based hormone-binding assay

GR-LBD hormone binding assays were performed as mentioned before (36) with some modifications. Experiments were performed in 40 mM HEPES, pH 7.5, 150 mM KCl, 5 mM MgCl2, 1 mM TCEP.

For steady-state hormone binding experiments, 1 μM GR-LBD was incubated with 10 μM Hsp70, 2 μM Ydj1, and 5 mM ATP for 1 h at room temperature. This was followed by adding 12 μM Hsp90 or Hsp90 phosphomimic with or without 20 μM co-chaperones like Hop and p23. After 45 min, 100 nM of fluorescein-labeled hormone Dexamethasone was added to the sample, and anisotropy was measured in a PHERAStar^Plus^ plate-reader (BMG LabTech) at an excitation of 485 nm and emission of 520 nm.

The K_D_s of the GR-LBD for different Hsp90 phosphomimic were measured by recording the fluorescence polarization of 0.5 μM Atto-488 labeled GR-LBD against a concentration gradient between 0.1 to 10 μM Hsp90. The data was fitted using Hill1 equation in OriginPro to calculate the dissociation constant.

### Analytical ultracentrifugation (AUC)

The multi-chaperone complexes with the GR-LBD were detected with randomly Atto-488 maleimide labeled 0.5 μM GR-LBD. They were first incubated with 10 μM Hsp70, 2 μM Ydj1 and 5 mM ATP in 40 mM HEPES, pH 7.5, 150 mM KCl and 5 mM MgCl_2_, 1 mM DTT for 1 h at 25 °C. This was followed by the addition of 12 μM Hsp90 and 20 μM of other co-chaperones. The reactions were further incubated for another 1 h at 25 °C and allowed to reach steady state. AUC was measured in an An-50 Ti rotor (Beckman) using ProteomLab Beckman XL-A centrifuge (Beckman Coulter) containing the AVIV fluorescence detection system (Aviv Inc). For all measurements, a rotor speed of 42,000 rpm was used. Data analysis was performed using Sedfit, Sedview (123, 124), and OriginPro.

## Data availability

All the data relevant to this work has been included in the article and the supporting information. All molecular dynamics input and topology files used for this study have been deposited in the Zenodo repository (https://doi.org/10.5281/zenodo.20539747). Further information and requests for resources should be directed towards the corresponding author, Johannes Buchner (johannes.buchner@tum.de).

## Supporting information

This article contains supporting information.

## Conflict of interest

The authors declare that they have no conflicts of interest with the contents of this article.
