# Supplementary material for: An isotype-specific phosphorylation of Hsp90 rewires co-chaperone regulations
Source: J Biol Chem. 2026 Jun 23;302(8):113292. doi: 10.1016/j.jbc.2026.113292 (PMC13400271; doi:10.1016/j.jbc.2026.113292)
Supplement: Supporting information [file mmc1.docx]

**An isotype-specific phosphorylation of Hsp90 rewires**

**co-chaperone regulations**

Supporting information

Tisya Banerjee^1^, Elisabetta Moroni^2^, Maximilian Riedl^1^, Giorgio Colombo^3^ and Johannes Buchner^1#^

^1^ Center for Protein Assemblies and Department of Bioscience, School of Natural Sciences, Technical University Munich, Ernst-Otto-Fischer Strasse 8, 85748 Garching, Germany.

^2^ Institute of Chemical Science and Technologies, Italian National Research Council (SCITEC-CNR), Milan, Italy

^3^ Department of Chemistry, University of Pavia, Pavia, Italy

# Corresponding Author: Johannes Buchner; e-mail: [johannes.buchner@tum.de](mailto:johannes.buchner@tum.de)

Main contact: Johannes Buchner

## Supporting figures

**
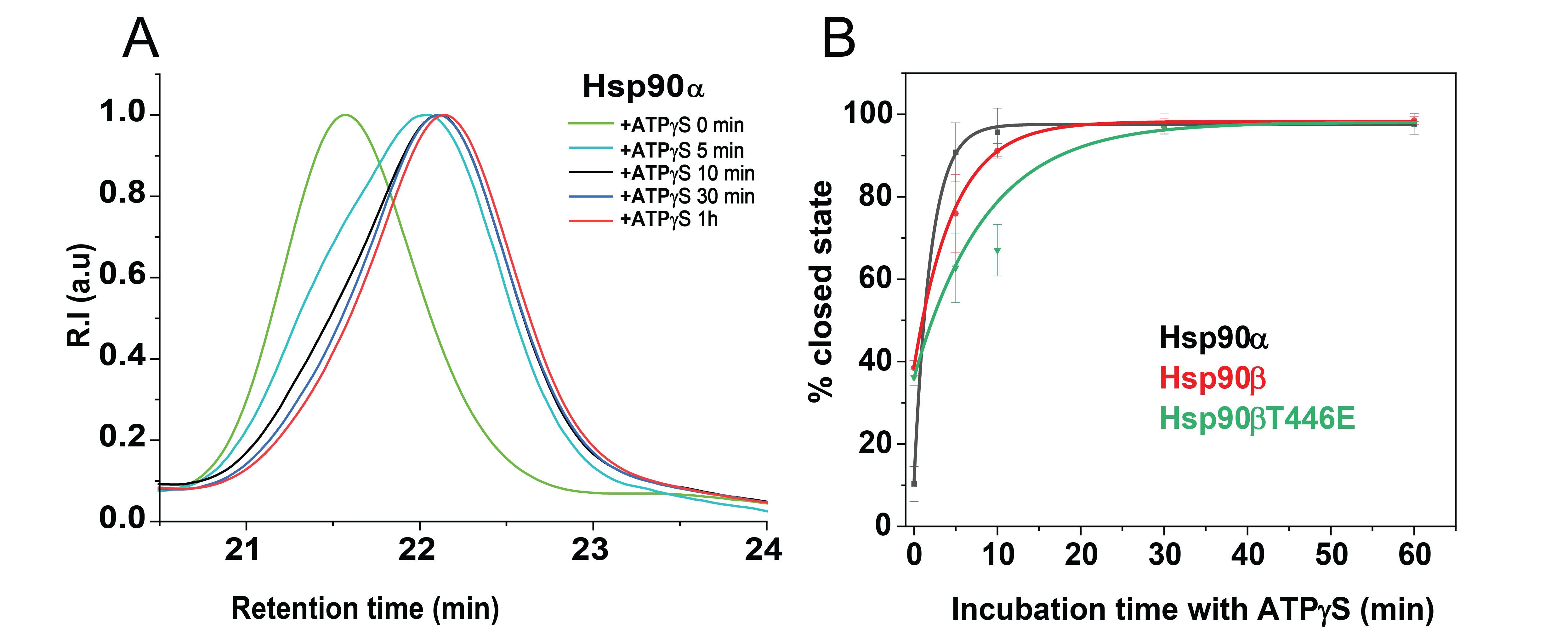
**

**Fig. S1.** **SEC-MALS profiles show difference in closing kinetics for different Hsp90 variants.**
**(A)** SEC-MALS experiments with elution profiles showing refractive indices of Hsp90α incubated with ATPγS from 0 -1h time scale.
**(B)** The percent of closed state for each Hsp90 variant with Hsp90α in black, Hsp90β in red, and Hsp90βT446E in green. The percentage of Hsp90 in closed state is calculated based on the area under the curve obtained from the biGaussian fit on the SEC-MALS Refractive Index profiles. Data points with error bars in (B) represent means ± SD of three technical repeats.

**
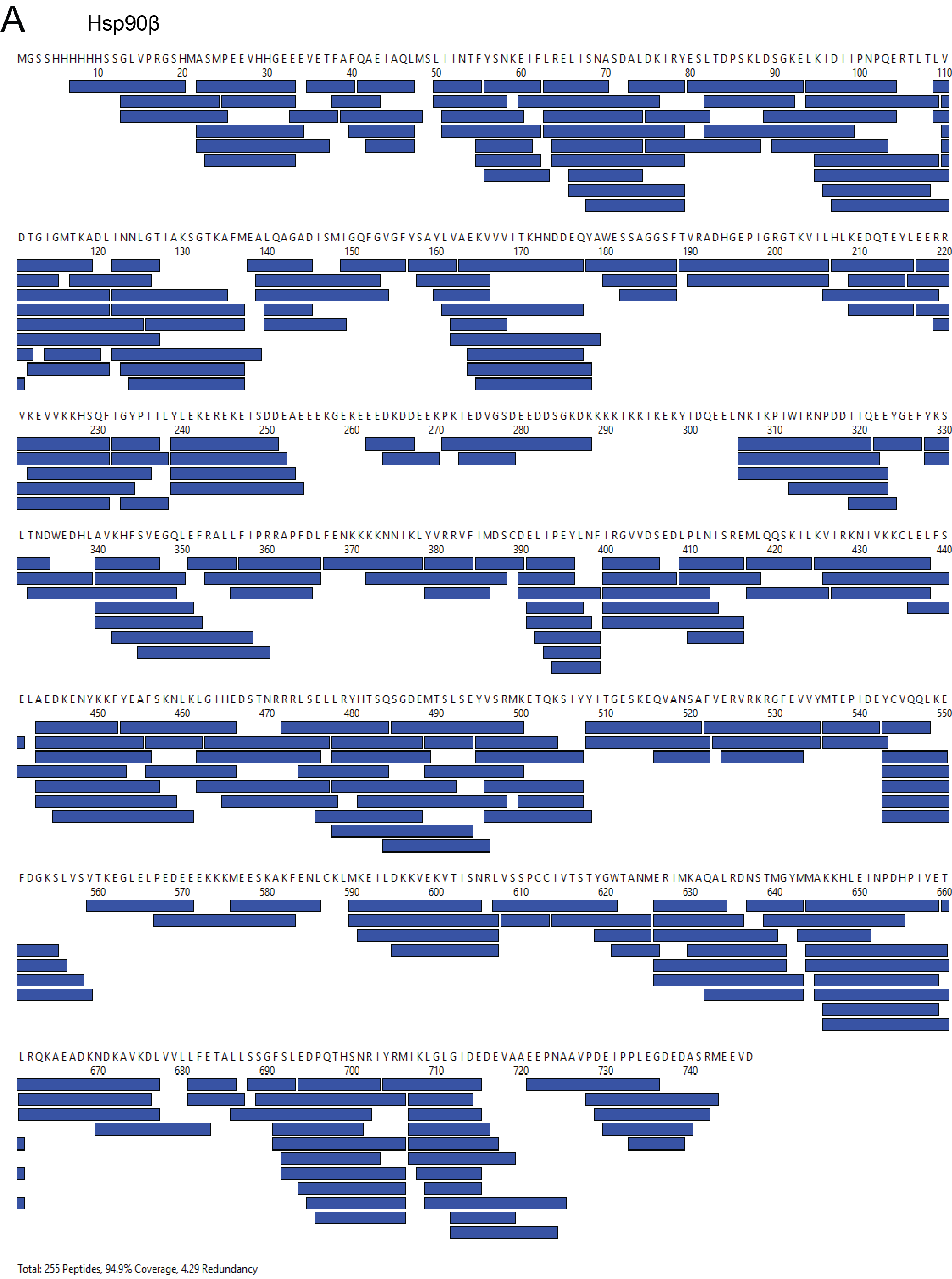
Fig S2**

**Fig. S2 (continued)**
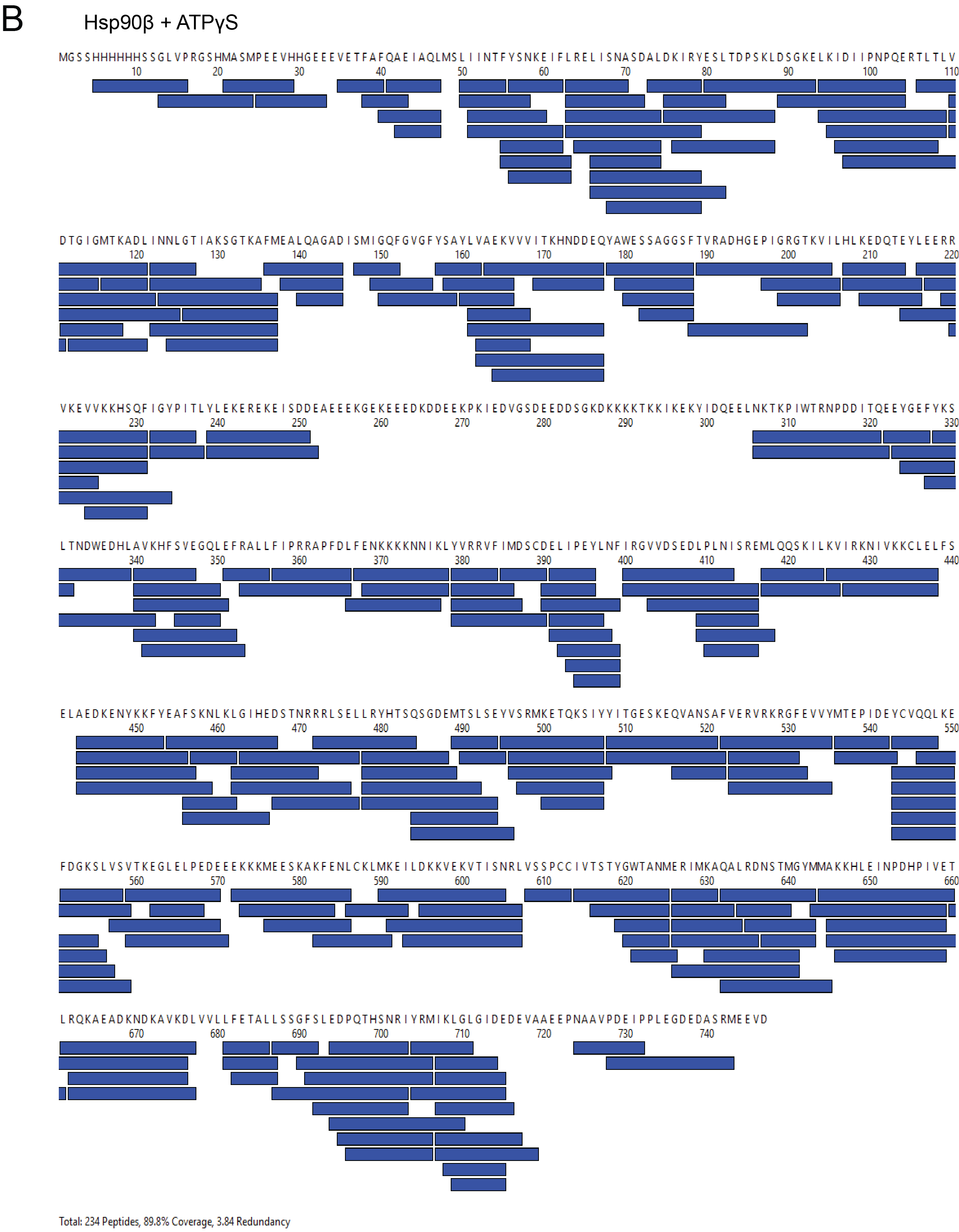


**Fig. S2 (continued)**
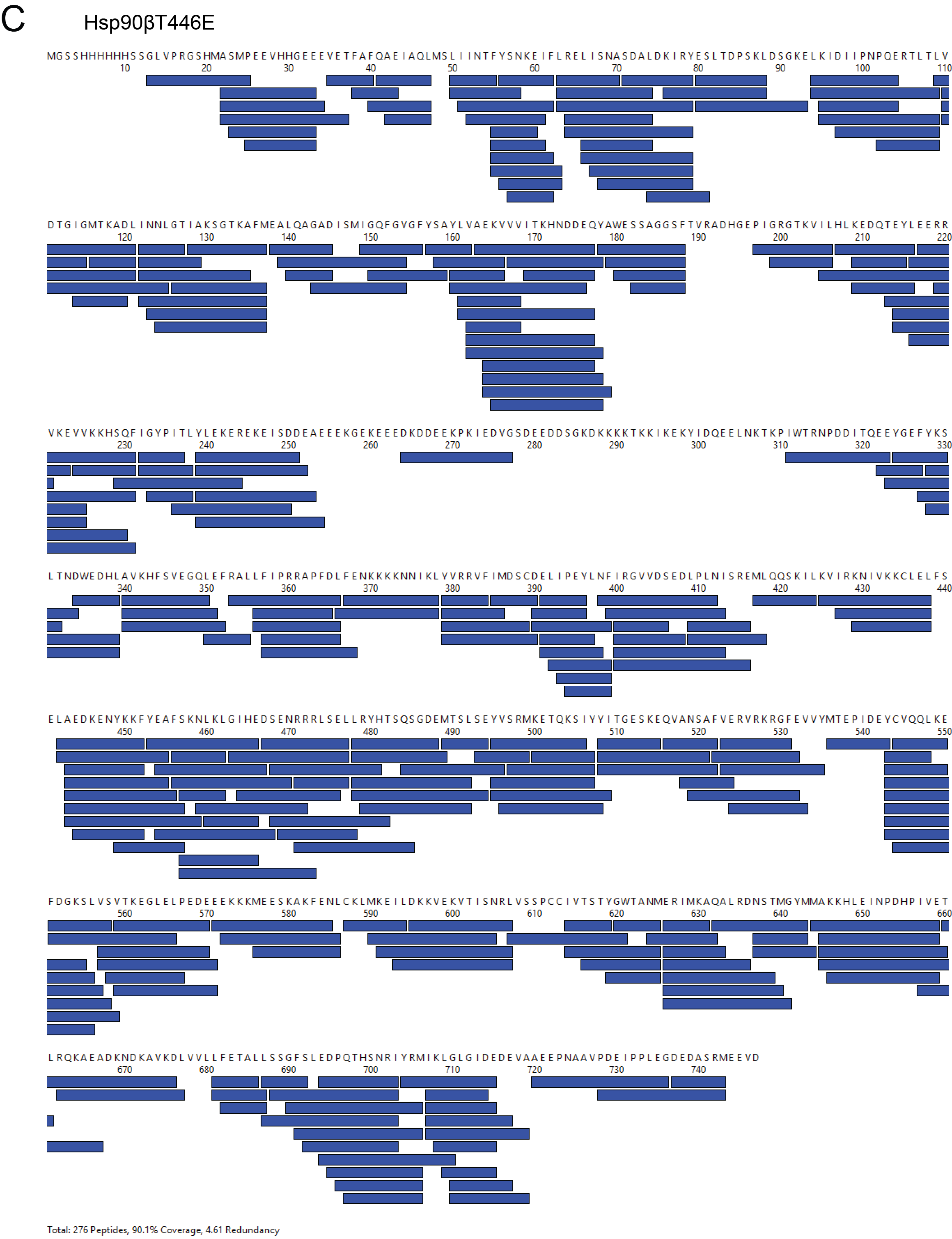


**Fig. S2 (continued)**
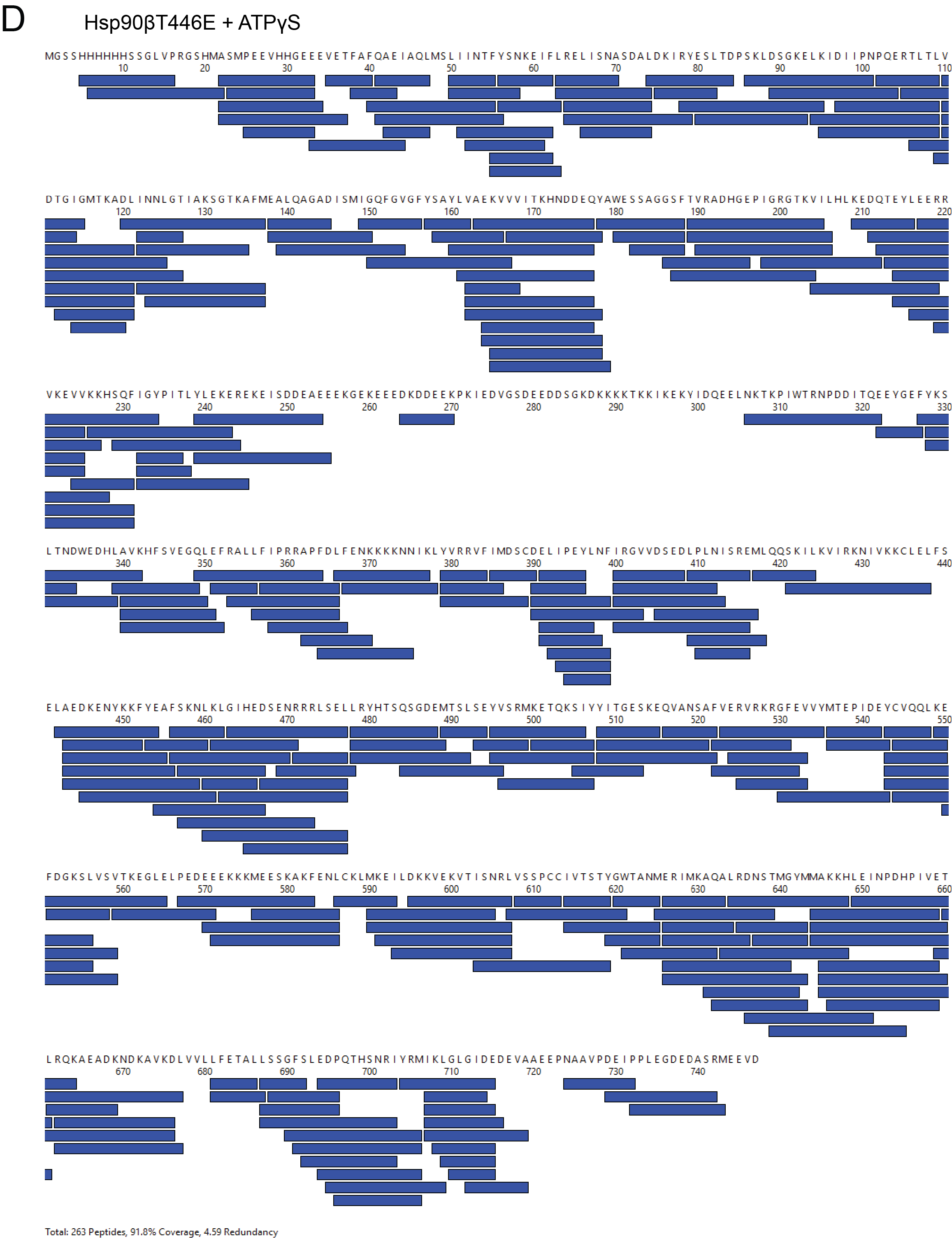


**Fig. S2. Peptide coverage of proteins (A) Hsp90β, (B) Hsp90β + ATPγS, (C) Hsp90βT446E, and (D) Hsp90βT446E + ATPγS used in HDX-MS in Fig. 2 (C-F)**


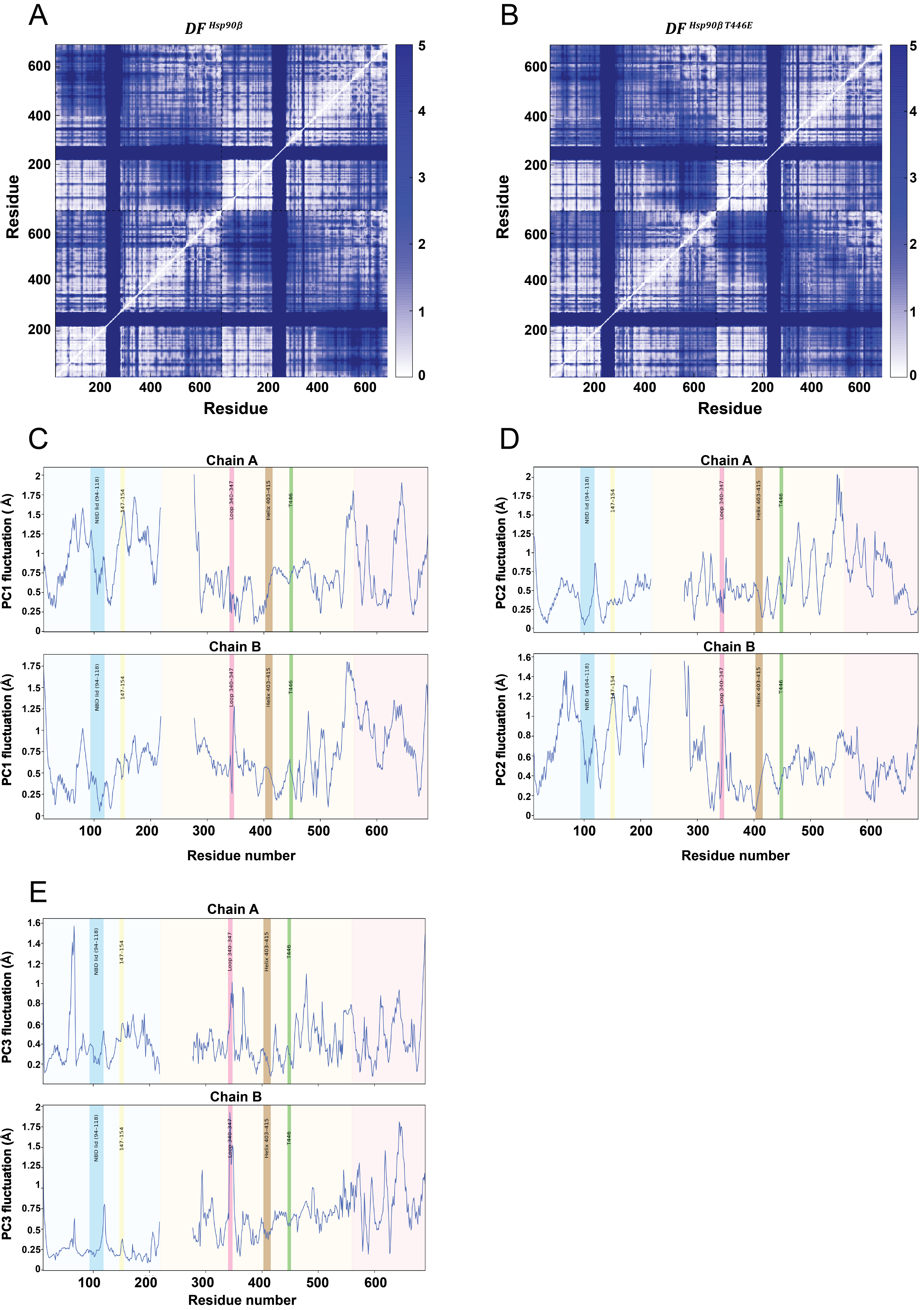

**Fig. S3. Structural dynamics analyses of Hsp90β and Hsp90βT446E MD simulations.**
**(A-B)** Residue-pair distance fluctuation (DF) matrices for Hsp90β (A) and Hsp90βT446E (B), computed from the concatenated replicates of each system as the time-averaged variance of C_α-_C_α_ distances.
**(C-E)** Per-residue RMS fluctuation profiles associated with PC1 (C), PC2 (D), and PC3 (E) obtained from the PCA performed on the concatenated trajectories of Hsp90β and Hsp90βT446E (backbone residues 220–277 excluded). RMS fluctuations were computed by back-projecting each principal component onto the PCA reference structure (time-averaged, fitted) so that higher values indicate residues contributing larger displacement amplitudes to the corresponding mode.

## Supporting tables

**Table S1: Top 10 hits obtained from Score Site tool on The Kinase Library for kinases that phosphorylate Hsp90β at T446 alongwith their kinase group (kinase family) and assigned scores (see Experimental Procedures)**

| **kinase** | **kinase_group** | **score_raw** | **score_log2** | **score_rank** |
| --- | --- | --- | --- | --- |
| **HRI** | **Other** | **32.01328721** | **5.000598919** | **1** |
| **EEF2K** | **Alpha** | **19.54936661** | **4.289049961** | **2** |
| **PERK** | **Other** | **17.91575852** | **4.163157221** | **3** |
| **TTK** | **Other** | **11.14278773** | **3.47803831** | **4** |
| **MLK4** | **TKL** | **10.42802965** | **3.382394684** | **5** |
| **ERK7** | **CMGC** | **9.152841743** | **3.194219736** | **6** |
| **PKR** | **Other** | **8.539300439** | **3.094117885** | **7** |
| **IRE2** | **Other** | **8.166278676** | **3.029678801** | **8** |
| **SRPK3** | **CMGC** | **6.895313523** | **2.785616151** | **9** |
| **TNIK** | **STE** | **6.792618361** | **2.7639678** | **10** |

**Table S2: Affinity of Hsp90 variants for co-chaperones (determined by SPR)**

| **Hsp90 variant** | **Hop (K_D_ in µM)** | **CHIP (K_D_ in µM)** | **Aha1 (K_D_ in µM)** | **p23 (K_D_ in µM)** |
| --- | --- | --- | --- | --- |
| **Hsp90α** | **1 ± 0.07** | **0.4 ± 0.09** | **0.2 ± 0.07** | **0.4 ± 0.2** |
| **Hsp90β** | **0.05 ± 0.02** | **1.5 ± 0.2** | **1.2 ± 0.8** | **2.9 ± 0.3** |
| **Hsp90βT446E** | **8.5 ± 1.5** | **0.6 ± 0.3** | **0.06 ± 0.01** | **3.7 ± 1.6** |

**Table S3: Affinity of Hsp90 variants for the GR-LBD (determined by anisotropy)**

| **Hsp90 variant** | **GR-LBD K_D_ (in µM)** |
| --- | --- |
| **Hsp90α** | **3.5 ± 0.9** |
| **Hsp90β** | **1.7 ± 0.6** |
| **Hsp90βT446E** | **1.6 ± 0.1** |
